# Knowledge and innovation communities, new potential for biomedical research in Europe

**DOI:** 10.1186/1878-5085-5-S1-A144

**Published:** 2014-02-11

**Authors:** Judita Kinkorova, Ondrej Topolcan

**Affiliations:** 1 Technology Centre Academy of Sciences, Prague, Czech Republic; 2 Medical Faculty Pilsen and Faculty Hospital Pilsen, Charles University Prague, Czech Republic

## 

The European Institute of Innovation and Technology (EIT) was based in 2008 in Budapest as a body of the European Union and its mission is “to Increase European sustainable growth and competitiveness by reinforcing the innovation capacity of the EU”. The aim is to enhance Europe’s ability to innovate, which translates into adapting quickly to the fast pace of development, being one step ahead in providing solutions to rapidly emerging societal problems and developing products that meet the demands and desires of consumers. The EIT is the first EU initiative to fully integrate all three sides of the Knowledge Triangle (higher education, research and business) by way of so-called Knowledge and Innovation Communities (KICs). The integration of all three sides and the effective transmission and sharing of knowledge, information and skills for joint exploitation is crucial to delivering the jobs and growth opportunities that Europe is seeking, as excellent researchers, students and entrepreneurs working in isolation are much less efficient in delivering the results needed and wanted by the market and consumers. EIT has been given an important role as a part of Horizon 2020, the framework programme for research and innovation for the period 2014 – 2020 with the objectives of addressing societal challenges e.g. health, demographic change, ageing population. In the EIT’s Strategic Innovation Agenda (SIA), the European Commission has presented a proposal that defines the framework for the EIT's operations for the period of 2014 to 2020:

A proposed budget of EUR 3.18 billion within the envelope of EUR 80 billion of Horizon 2020, the EU's future Framework Programme for Research and Innovation (around 3.5 % of the total Horizon 2020 budget). Creation of six new KICs is launched in two waves. Proposed themes (outlined in more detailed in the proposal for the EIT’s Strategic Innovation Agenda) and indicative timeline below:

**Figure 1:**
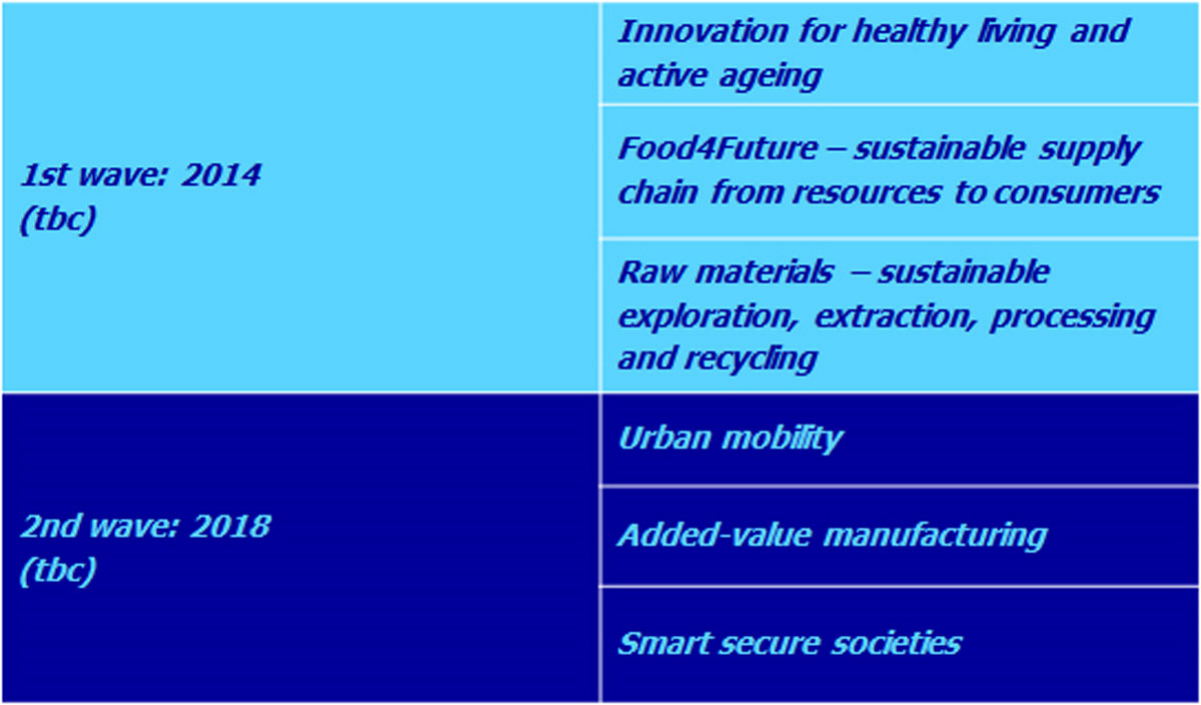


The proposed KIC “Innovation for healthy living and active ageing” for 2014 offers new and challenging opportunity for all actors in the “Health” area to take part in and to contribute to foster and enhance research and development in this field, and to be a member of a big excellent research family in Europe.

